# Recent Advances in Immunotherapy for Non-Muscle-Invasive Bladder Cancer

**DOI:** 10.3390/cancers18040623

**Published:** 2026-02-14

**Authors:** Abby L. Grier, Jeffrey Y. Zhong, Spyridon Basourakos, Adam Calaway, Parminder Singh, Yousef Zakharia, Fabrice Lucien, R. Jeffrey Karnes, Vidit Sharma, Paras Shah, Brian A. Costello, Lance C. Pagliaro, Jacob J. Orme, Jason R. Brown, Albert Jang

**Affiliations:** 1 School of Medicine, Case Western Reserve University School of Medicine, Cleveland, OH 44106, USA; abby.grier@case.edu; 2 Department of Medicine, University Hospitals Cleveland Medical Center, Case Western Reserve University School of Medicine, Cleveland, OH 44106, USA; jeffrey.zhong@uhhospitals.org; 3 Department of Urology, University Hospitals Cleveland Medical Center, Case Western Reserve University School of Medicine, Cleveland, OH 44106, USA; 4 Division of Hematology and Oncology, Department of Internal Medicine, Mayo Clinic, Phoenix, AZ 85054, USA; 5 Department of Urology, Mayo Clinic, Rochester, MN 55905, USA; 6 Division of Medical Oncology, Department of Oncology, Mayo Clinic, Rochester, MN 55905, USA; 7 Division of Solid Tumor Oncology, Department of Medicine, University Hospitals Cleveland Medical Center, Case Western Reserve University School of Medicine, Cleveland, OH 44106, USA; jason.brown3@uhhospitals.org; 8 Case Comprehensive Cancer Center, Case Western Reserve University, Cleveland, OH 44106, USA

**Keywords:** Bacillus Calmette-Guérin, bladder cancer, immune checkpoint inhibitor, immunotherapy, intravesical therapy, non-muscle-invasive bladder cancer, transurethral resection of bladder tumors

## Abstract

Non-muscle-invasive bladder cancer (NMIBC) comprises approximately 75% of new bladder cancer cases and generally carries a favorable prognosis, yet high rates of recurrence and progression necessitate close surveillance with frequent cystoscopies and repeated transurethral resections. Upfront treatment for high-risk disease is typically Bacillus Calmette-Guérin (BCG), although combinations with immune checkpoint inhibitors have reported results. Patients with BCG-unresponsive, intolerant, or refractory disease represent a subset of patients with high risk of progression, with early radical cystectomy being the standard approach for this setting. Global BCG shortages and the substantial impact of cystectomy on quality of life underscore the need for therapeutic alternatives. Over the past decade, investigational trials in immunotherapy have expanded treatment options for BCG-unresponsive NMIBC with CIS, leading to FDA approval of intravesical nadofaragene firadenovec, nogapendekin alfa-inbakicept, and systemic pembrolizumab. This narrative review summarizes developments in intravesical and systemic immunotherapies for NMIBC, highlights ongoing trials, and addresses controversies in trial design, treatment sequencing, comparative efficacy, and safety.

## Simple Summary

Bladder cancer confined to the lining usually has a good prognosis, but it often returns or becomes more aggressive. Standard treatment involves repeated procedures to remove tumors and placing weakened bacteria into the bladder to activate the immune system. Some patients whose cancers do not respond to standard treatment may need bladder removal, which can greatly affect quality of life. Over the past decade, several immune-based treatments have been developed and approved, including therapies delivered into the bladder or given through the bloodstream. This narrative review summarizes these advances, ongoing studies, and the challenges in deciding the best treatment approach for these patients.

## 1. Introduction

Non-muscle-invasive bladder cancer (NMIBC) encompasses tumors confined to the bladder mucosa or lamina propria, including non-invasive papillary (Ta), invading the lamina propria (T1), and carcinoma in situ (CIS). CIS is considered a distinct cohort from papillary-only tumors owing to fundamental differences in morphology, mucosal involvement, multifocality, ability to visualize on cystoscopy, endoscopic resectability, higher intrinsic risk for progression to muscle invasive bladder cancer (MIBC), and therapeutic response. NMIBC represents approximately 75% of new bladder cancers and generally confers a favorable prognosis, with 5-year survival exceeding 90% [1,2]. However, recurrence and progression to MIBC are common, necessitating close surveillance with frequent cystoscopies and repeated transurethral resections of bladder tumors (TURBT), imposing a significant burden on the patient and healthcare system.

The American Urological Association (AUA), the European Association of Urology (EAU), and the Society of Urologic Oncology (SUO) established stratification guidelines for NMIBC risk of progression [3,4]. However, these organizations categorize heterogenous clinicopathologic features differently, complicating clinical decision-making and trial design. The EAU uses a four-tiered model with a category for very high-risk disease, while the AUA and SUO frameworks are three-tiered; criteria such as age, prostatic urethral involvement, and variant histology are weighed differently across systems. Bacillus Calmette-Guérin (BCG) responsiveness is incorporated into risk assessment across guidelines, as tumors unresponsive to BCG represent an especially high-risk subset unlikely to benefit from further intravesical therapy [5].

As defined by the First International Consultation on Bladder Tumors, BCG failure can be divided into four categories: BCG-refractory, BCG-relapsing, BCG-unresponsive, and BCG-intolerant NMIBC. BCG-refractory NMIBC is defined as persistent high-grade disease at 6 months after adequate BCG therapy or stage/grade progression by 3 months after the first cycle despite adequate BCG. BCG-relapsing NMIBC is the recurrence of high-grade disease within 6 months of adequate BCG treatment, after achieving an initial response to BCG. BCG-unresponsive NMIBC is used to describe both BCG-refractory and BCG-relapsing disease. In comparison, BCG-intolerant NMIBC indicates an inability to receive adequate BCG due to treatment-related toxicity [6]. Adequate BCG is defined as ≥5/6 induction doses plus either ≥2/3 maintenance doses or ≥2/6 doses of a second induction course [4]. Due to the indolent nature of low-risk NMIBC, after being histologically confirmed following TURBT, patients are managed with surveillance via scheduled cystoscopies, which may be de-escalated over time to minimize morbidity following shared decision-making [7]. For patients with intermediate-risk Ta/T1 NMIBC, standard of care is TURBT followed by a single post-operative instillation of intravesical chemotherapy, with subsequent consideration of either adjuvant intravesical chemotherapy or maintenance BCG for at least one year, as tolerated [8]. In contrast, high-risk NMIBC requires aggressive management, typically TURBT with induction and up to three years of maintenance BCG [3,4]. Radical cystectomy has been recommended for BCG-unresponsive disease, but its significant impact on quality of life fuels current investigation into bladder-sparing alternatives.

Bladder cancer is characterized by high tumor mutational burden, often exceeding that of other immunologically active tumors such as melanoma or non-small-cell lung cancer [9]. Many bladder tumors have an APOBEC mutational signature that enhances immunogenicity and leads to the generation of high neoantigen loads [10]. Therefore, there is strong biological rationale for immune stimulation with BCG and other novel immunotherapy approaches. This narrative review summarizes recent advances in intravesical and systemic immunotherapy approaches for NMIBC, incorporating published literature through October 2025 and key data presented at ESMO 2025, which together hold potential to inform the future standard of care.

## 2. BCG and Potential Pitfalls

BCG, an attenuated strain of the bacteria *Mycobacterium bovis*, was first harnessed for the treatment of NMIBC in 1976 [11]. This advancement was built upon the observation that patients with active tuberculosis lesions had a lower incidence of cancer at autopsy [12]. In the initial study, administering an induction course of six weekly BCG instillations to nine patients resulted in a marked reduction in bladder cancer recurrence compared with historical outcomes [11].

This groundbreaking work prompted further investigation of intravesical BCG for NMIBC. Two randomized controlled trials (RCT) in the early 1980s demonstrated statistically significant reduction in recurrence, decreased disease progression, and durable benefit with TURBT and BCG versus TURBT alone [13,14]. A study in 262 patients with rapidly recurrent NMIBC demonstrated the clear superiority of BCG over doxorubicin and supported the 1990 United States Food and Drug Administration (FDA) approval of BCG for stage Ta, T1, and CIS bladder cancers [15,16]. Despite the well-established efficacy of BCG in NMIBC, a substantial subset will not respond to therapy or will experience disease recurrence. Across all patients with intermediate- and high-risk disease who receive intravesical BCG, approximately one third have primary refractory disease. Among those who do achieve an initial complete response (CR) to BCG, defined as absence of detectable disease at protocol-specified reassessment following treatment, approximately 40% will have tumor recurrence within 3 years of therapy and approximately 15% will progress to MIBC despite initial response [17,18].

Over the last decade, there has been a persistent BCG shortage complicating the care of patients with NMIBC, leading to shorter treatment regimens or the inability to receive BCG altogether, prompting investigation into alternatives (Table 1). One such alternative is the strain of BCG. The Connaught strain has been found to reduce recurrence relative to the TICE strain [19]. This finding prompted head-to-head comparisons of the remaining BCG strains, including the ongoing SWOG S1602 trial (NCT03091660) evaluating the Tokyo-172 and TICE strains [20]. One promising regimen requiring less BCG without compromising efficacy has been the combination of BCG with mitomycin. The phase III ANZUP 1301 trial (NCT02948543) investigated this combination versus BCG alone in 501 patients with high-risk NMIBC (pTa/pT1 ± CIS) following TURBT, demonstrating no difference in disease-free survival (DFS), defined as any recurrence or progression of disease from treatment initiation, at 24 months (HR 0.86, 95% CI = 0.64–1.14) [21]. Alternatively, the BRIDGE trial (NCT05538663) is investigating intravesical docetaxel and gemcitabine versus BCG in 870 patients with BCG-naïve high-grade NMIBC [22]. Despite BCG’s long-standing position as the standard of care, its incomplete efficacy and ongoing barriers to access highlight a critical unmet need to expand the armamentarium of immunotherapies in NMIBC.

## 3. Immune Activation via Intravesical Therapies

Intravesical immune-activating therapies have gained increasing attention as effective bladder-sparing options amid recurrent BCG shortages. These agents stimulate potent local immune responses, leveraging innate and adaptive pathways to induce tumor clearance while minimizing systemic toxicity. A diverse pipeline of investigational therapies is now emerging, with each employing distinct mechanisms to enhance local immune engagement and improve outcomes across both BCG-naïve and BCG-unresponsive NMIBC (Table 2).

### 3.1. TARA-002

TARA-002 is formulated from inactivated Streptococcus pyogenes (Su strain), exploiting its immune-stimulating capabilities. While similar to intravesical BCG, TARA-002 has a different mechanism, and is internalized into urothelial cancer cells via a separate toll-like receptor 2-dependent pathway that activates the innate immune system [39]. This localized innate immune response recruits cytotoxic T cells, resulting in combined direct cytotoxicity and systemic immune responses which further promote anti-tumor activity. Following initial signs of efficacy in the phase Ia/Ib ADVANCED-1 trial (NCT05085990), the phase II ADVANCED-2 trial (NCT05951179) is investigating the safety and efficacy of TARA-002 in patients with BCG-naïve or BCG-unresponsive high-grade NMIBC (Ta/T1 or CIS) [23,24]. Preliminary pooled data from ADVANCED-1 and ADVANCED-2 showed 38% CR at 3 months, including 63% CR at 3 months in patients with CIS. The treatment was well tolerated with no grade 3 treatment-related adverse events (TRAEs) [25].

### 3.2. Nogapendekin Alfa-Inbakicept

Nogapendekin alfa-inbakicept selectively stimulates cytotoxic CD8+ T cells and natural killer cells by acting as an interleukin (IL)-15 superagonist. This induces a robust local antitumor response that works synergistically with the innate immune system and effector T-cell activation of BCG. The QUILT 3.032 phase II/III trial (NCT03022825) assessed nogapendekin alfa-inbakicept in combination with BCG (Cohort A) in patients with BCG-unresponsive NMIBC (CIS ± Ta/T1). Another cohort investigated nogapendekin alfa-inbakicept in combination with BCG in a subset of patients with high-grade Ta/T1 papillary NMIBC (Cohort B) [26,27]. In Cohort A, 71% of patients had CR when assessed 3 months after induction intravesical therapy (95% CI = 61.1–79.6%). Cystectomy-free rate at 36 months was 84%. Prior subgroup analysis showed that CR rates of patients with CIS alone, CIS/Ta disease, and CIS/T1 disease were 68% (95% CI = 54.8–80.1%), 81% (95% CI = 54.4–96.0%), and 67% (95% CI = 29.9–92.5%) respectively [26]. The 80 patients in Cohort B had a median DFS of 25.3 months with a cystectomy-free rate of 82% at 36 months. Overall, nogapendekin alfa-inbakicept in combination with BCG was well tolerated with TRAEs consistent with known toxicities of intravesical BCG. Nogapendekin alfa-inbakicept in combination with BCG for patients with BCG-unresponsive NMIBC with CIS ± Ta/T1 papillary disease was approved by the FDA as a breakthrough therapy in April 2024 as a possible alternative to cystectomy [40].

A phase II clinical trial (NCT06829823) is examining the efficacy of nogapendekin alfa-inbakicept for BCG-naïve, intermediate-risk Ta/T1 NMIBC in combination with BCG or gemcitabine as ablative therapy without surgical intervention [28]. CR rate will be assessed at month 3 or month 6 for re-inducted patients. If these ablative therapies show promise, it could offer an alternative to surgical intervention in select patients.

### 3.3. Nadofaragene Firadenovec

Nadofaragene firadenovec is an adenoviral vector-based gene therapy that delivers human interferon alfa-2b (IFNα2b) to urothelial cells. This induces direct cytotoxicity mediated by Tumor Necrosis Factor (TNF)-Related Apoptosis-Inducing Ligand (TRAIL)-dependent pathways and indirectly enhances both innate and adaptive immune responses to favor immunogenic cell death [41,42]. A phase III clinical trial (NCT02773849) included 151 patients with BCG-unresponsive NMIBC (high-grade Ta/T1 or CIS ± Ta/T1). Among patients with CIS, 53.4% had CR at 3 months (95% CI = 43.3–63.3%), and 24.3% remained high-grade recurrence-free at 12 months (95% CI = 16.4–33.7%). Among patients with CIS and initial CR, 45.5% had high-grade recurrence-free survival, defined as no high-grade recurrence detected following treatment initiation, at 12 months [29]. In patients with high-grade Ta/T1 NMIBC without CIS, 72.9% of patients were high-grade recurrence-free at 3 months (95% CI = 58.2–84.7%), and the median duration of response (DOR) was 12.35 months. Recurrence-free survival, defined as the time from treatment initiation to the first disease recurrence at 12 months, occurred in 43.8% of patients (95% CI = 29.5–58.8%). Overall, 26% of patients had undergone radical cystectomy at 12 months. Most patients experienced mild and transient grade 1–2 TRAEs, while 6 patients experienced grade 3 TRAEs. Following these promising results, the FDA granted approval of nadofaragene firadenovec as monotherapy in 2022 for the treatment of BCG-unresponsive, high-risk NMIBC with CIS ± Ta/T1 papillary disease [43].

Given the positive results of this phase III study, several trials have been initiated to evaluate nadofaragene firadenovec in combination regimens, across broader clinical settings, and in direct comparison with other standard treatments for NMIBC. The randomized phase II ABLE-22 trial (NCT06545955) is comparing the efficacy of nadofaragene firadenovec as monotherapy or in combination with either intravesical gemcitabine and docetaxel or pembrolizumab versus nadofaragene firadenovec monotherapy. Target enrollment is 150 patients with BCG-unresponsive NMIBC (CIS ± high grade Ta/T1). The primary endpoint is CR rate at 3 months or at 6 months with re-induction if no CR was achieved at 3 months. Secondary endpoints include DOR, incidence of muscle-invasive progression, cystectomy-free survival, pathological staging, OS, and safety [30]. The phase III ABLE-32 trial (NCT06510374) aims to recruit 454 patients with intermediate-risk NMIBC to examine the efficacy of nadofaragene firadenovec versus observation alone, using recurrence-free survival as the primary endpoint [31]. COMPARE IT (NCT06929286) is a phase III trial evaluating the efficacy of nadofaragene firadenovec versus other non-cystectomy treatments for high-grade NMIBC, with a primary endpoint of high-grade recurrence-free survival [32].

### 3.4. Cretostimogene Grenadenorepvec

Cretostimogene grenadenorepvec (CG0070) is a dual-mechanism viral therapy that utilizes a modified adenovirus to selectively target bladder cancer cells with alterations in the retinoblastoma tumor suppressor gene. Following entrance into the cancer cell, cretostimogene grenadenorepvec replication directly lyses the cancer cell. The viral vector carries a transgene for granulocyte macrophage colony-stimulating factor (GM-CSF), expression of which promotes local and systemic antitumor immune responses [44]. The BOND-002 phase II clinical trial (NCT02365818) investigated the efficacy of cretostimogene grenadenorepvec in 45 patients with NMIBC (high-grade Ta/T1 or CIS ± Ta/T1) with failed BCG induction therapy or recurrence following BCG. The 6-month CR rate was 47% (95% CI = 32–62%) on interim analysis [33]. On subset analysis, there was 50% 6-month CR (95% CI = 33–67%) in patients with CIS and 33% 6-month CR (95% CI = 8–70%) in patients with pure Ta/T1 NMIBC. The treatment was well tolerated, with only grade 1–3 TRAEs reported.

BOND-003 (NCT04452591) was initiated following the promising interim results of BOND-002. This single-arm, open-label, phase III clinical trial is evaluating the efficacy of cretostimogene grenadenorepvec monotherapy in patients with BCG-unresponsive high-risk NMIBC. Cohort C consists of 115 patients with CIS ± high-grade Ta/T1 papillary disease, while Cohort P will enroll 75 patients with high-grade Ta/T1 papillary disease only. Interim analysis of Cohort C demonstrated 74.5% CR rate (95% CI = 65.4–82.4%), 53.8% of repeat induction patients converting to CR, and 92.4% cystectomy-free survival rate [34]. Durable responses were observed in 45% of patients at 6 months, 25% of patients at 12 months, and 12% of patients at 21 months. Cretostimogene grenadenorepvec was well-tolerated; all TRAEs were grades 1–2, with no grade 3 TRAEs reported. These encouraging interim results led to FDA Fast Track designation in 2023 for cretostimogene grenadenorepvec monotherapy in patients with high-risk BCG-unresponsive CIS ± Ta/T1 papillary disease [45].

Combination treatment with cretostimogene grenadenorepvec and pembrolizumab was evaluated in the phase II CORE-001 clinical trial (NCT04387461) in patients with BCG-unresponsive NMIBC with CIS. Among the 35 patients included in intention-to-treat analysis, 83% achieved CR at 3 months (95% CI = 70.4–95.3%), and 51.4% maintained CR at 24 months (95% CI = 34.9–68.0%). Notably, no patients had disease progression to MIBC at the time of data cutoff. The regimen was well tolerated overall, as no patients developed grade 3 TRAEs related to cretostimogene grenadenorepvec, while grade 3 TRAEs related to pembrolizumab occurred in 14.3% of patients [35].

The potential role of cretostimogene grenadenorepvec beyond BCG-unresponsive NMIBC is also under active investigation. CORE-008 (NCT06567743) is an ongoing phase II trial evaluating the efficacy of cretostimogene grenadenorepvec in patients with high-risk NMIBC (high-grade Ta/T1 or CIS ± Ta/T1) who are BCG-naïve (cohort A) or BCG-exposed (cohort B). Cohorts A and B will not be compared. The primary endpoints are CR at any time for patients with CIS and high-grade event-free survival for patients with papillary-only disease [36]. In addition, the phase III PIVOT-006 (NCT06111235) trial is assessing adjuvant cretostimogene grenadenorepvec for intermediate-risk NMIBC following TURBT versus TURBT alone. The primary endpoint is recurrence-free survival, and secondary endpoints include safety, tolerability, progression-free survival, and time to next intervention [37].

### 3.5. Detalimogene Voraplasmid

Detalimogene voraplasmid is a non-viral, non-integrating, gene-based therapy that delivers a plasmid encoding both IL-12 and synthetic RNA sequences designed to activate retinoic acid-inducible gene I (RIG-I). Activation of RIG-I, together with IL-12 expression, generates robust anti-tumor activity by stimulating both innate and adaptive immune responses. Its safety and efficacy in the setting of BCG-unresponsive NMIBC with CIS are being evaluated in the phase I/II LEGEND trial (NCT04752722). In the phase I cohort, overall CR was 73%. The phase II portion of LEGEND has shown promising preliminary results, with a 3-month CR rate of 67%, 6-month CR rate of 47%, and an overall CR rate of 71% [38]. All TRAEs were grade 1–2, with dysuria (21.4%), bladder spasms (19.0%), pollakiuria (11.9%), and fatigue (11.9%) being the most common. A separate cohort of BCG-naïve patients, or patients inadequately treated with BCG, will also be enrolled

## 4. Systemic Immunotherapy

High PD-1 positivity is associated with significantly lower 5-year recurrence-free and progression-free survival after BCG [46]. BCG works by inducing a local inflammatory response; thus, PD-(L)1 checkpoint blockade is a rational option to restore antitumor T-cell activity and enhance responsiveness to BCG or subsequent therapies after BCG failure. Systemic checkpoint inhibition is under active investigation in NMIBC across three treatment settings: following BCG failure, in combination with BCG, and as a potential alternative to BCG (Table 3).

### 4.1. Systemic Immunotherapy After BCG Failure

Pembrolizumab, a PD-1 inhibitor, became the first FDA-approved systemic immunotherapy for BCG-unresponsive, high-risk NMIBC (CIS ± Ta/T1 papillary disease) in patients declining or ineligible for cystectomy following the positive results of the phase II KEYNOTE-057 trial (NCT02625961) in 2020 [59]. In cohort A (CIS ± papillary tumors), median follow-up was 36.4 months, and 96 patients were included in the efficacy analysis. The 3-month CR rate was 41% (95% CI = 30.7–51.1%), exceeding that of previously approved intravesical salvage therapies. Response was durable, with a median CR duration of 16.2 months (95% CI = 6.7–36.2%) and 46% of initial responders maintaining CR at 12 months. The safety profile of pembrolizumab was consistent with previously reported toxicities, with 13% of patients experiencing grade ≥ 3 TRAEs and no treatment-related deaths [47].

Cohort B (Ta/T1 papillary tumors without CIS) of KEYNOTE-057 enrolled 132 patients with median follow-up of 45.4 months. The 12-month DFS rate was 43.5% (95% CI = 34.9–51.9%) with a median DFS of 7.7 months (95% CI = 5.5–13.6). At 12 months, 83% of patients remained free from progression to higher grade, higher stage, or death; 97% had not developed MIBC or metastatic disease. These positive findings expanded the potential role of pembrolizumab to patients without CIS [47]. Post hoc analyses pooling the A and B cohorts demonstrated that delayed radical cystectomy following non-response to pembrolizumab was not associated with inferior clinical or pathological outcomes, supporting the safety of systemic immunotherapy even when not effective as a bladder-sparing approach [48]. Cohort C is evaluating pembrolizumab combinations with novel checkpoint inhibitors vibostolimab, targeting TIGIT, or favezelimab, targeting LAG3. The results for this cohort are pending.

Atezolizumab, a PD-L1 inhibitor, was evaluated in the single-arm, multicenter phase II SWOG S1605 trial (NCT02844816) among a similar patient population. A total of 129 patients with BCG-unresponsive high-risk (high-grade Ta, T1, or CIS) NMIBC were included in the efficacy analysis. The primary endpoint was the pathological CR rate determined via mandatory biopsy at 6 months. The trial did not meet its prespecified efficacy threshold of a CR rate of 30% at 6 months in the CIS group, which was 27% for 74 patients with CIS. Atezolizumab demonstrated a median CR duration of 17 months, and 56% (95% CI = 34–77%) of patients had at least 12 months of durable response. There was a significant toxicity profile, including grade ≥3 TRAEs in 16% of patients and three treatment-related deaths [49].

ADAPT-BLADDER (NCT03317158) was a multicenter phase I trial investigating the PD-L1 inhibitor durvalumab as monotherapy or in combination with other treatment modalities. Patients with high-grade Ta, T1, or CIS BCG-unresponsive NMIBC were assigned to durvalumab monotherapy (*n* = 3) or in combination with BCG (*n* = 13), among other cohorts. Durvalumab combination with BCG showed the most promising activity, with a 3-month CR rate of 85% and 73% maintaining CR at 12 months. In contrast, durvalumab monotherapy yielded CR in only 33% of patients at 3 months, with 0% maintaining durable response at 12 months. The EBRT combination cohort had intermediate efficacy, with a 3-month CR rate of 50% and 12-month CR rate of 33%. With a median follow-up of 20.6 months, no unexpected safety signals were observed [50].

The phase Ib/II trial DURANCE (NCT04106115) evaluated durvalumab in combination with S-488210/S-488211, a 5-peptide cancer vaccine, in 14 patients with BCG-unresponsive (resistant or relapsing) or BCG-intolerant NMIBC (Ta 14%, 43% T1, 43% CIS). Preliminary phase Ib data demonstrated favorable tolerability, with no grade ≥ 3 TRAEs or dose-limiting toxicities. The most common adverse events were grade 1 injection site reactions (50%) and pruritis (14.3%). At data cutoff, 3- and 6-month DFS rates were 71% and 57%, respectively. These early findings support potential antitumor activity without significant additive toxicity. Phase II recruitment is ongoing [51].

### 4.2. Systemic Immunotherapy in Combination with BCG

POTOMAC (NCT03528694) is a phase III trial investigating durvalumab in combination with BCG in 1018 BCG-naïve patients with high-risk NMIBC (high-grade Ta or T1 ± CIS). Patients were randomized 1:1:1 to BCG alone, durvalumab with BCG induction only, or durvalumab with BCG induction and maintenance. With a median follow-up of 60.7 months, 24-month DFS was 86.5% (95% CI = 82.2–89.8%) in the durvalumab plus BCG induction and maintenance arm versus 81.6% (95% CI = 76.9–85.3%) with BCG alone (HR 0.68, 95% CI = 0.50–0.93; *p* = 0.02). OS did not differ significantly between these two groups (HR 0.80, 95% CI = 0.53–1.20). However, the addition of durvalumab was associated with increased toxicity, as 21% of patients had grade 3–4 TRAEs in the durvalumab with BCG induction and maintenance group compared to 4% with BCG alone. The most common grade 3–4 TRAEs were dysuria and increased lipase. While a positive study, the high toxicity risk may counteract any benefit of adding durvalumab to BCG induction and maintenance [52].

PATAPSCO (NCT05943106) is a phase IIIb single-arm study evaluating durvalumab plus BCG induction and maintenance in 100 patients with high-risk NMIBC (high grade Ta/T1 ± CIS), and is expected to provide real-world context into this, reflecting U.S. national practice patterns, BCG availability, and patient demographics that may differ from those reported in the global POTOMAC trial.

CREST (NCT04165317) is a phase III trial evaluating sasanlimab, a subcutaneous PD-1 inhibitor, in 1055 patients with high-risk NMIBC (high-grade Ta or T1 ± CIS). Patients were randomized 1:1:1 to receive sasanlimab with BCG induction and maintenance, sasanlimab with BCG induction only, or BCG alone. The primary endpoint was event-free survival (EFS). At a median follow-up of 36.3 months, the addition of sasanlimab to BCG induction and maintenance reduced the risk of events by 32% relative to BCG alone (HR 0.68, 95% CI = 0.49–0.94; *p* = 0.01). Benefit was most pronounced in patients with CIS (HR 0.53, 95% CI = 0.29–0.98), while patients without CIS did not appear to have as much benefit (HR 0.76, 95% CI = 0.52–1.11). When sasanlimab was combined with only BCG induction, there was no improvement in outcomes over BCG alone (HR 1.16, 95% CI = 0.87–1.55; *p* = 0.84), reinforcing the importance of maintenance BCG. Patients in the control arm lacked access to immunotherapy post-recurrence or progression, limiting the interpretation of CREST outcomes. Safety was manageable with no new safety signals reported for these agents, with 29.1% patients having grade ≥3 TRAEs with sasanlimab compared to 6.3% without sasanlimab [54].

ALBAN (NCT03799835) is a phase III trial investigating atezolizumab in combination with BCG in BCG-naïve patients with high-risk NMIBC (high grade Ta or T1 ± CIS), with 517 patients randomized 1:1 to receive BCG plus atezolizumab versus BCG alone. EFS was the primary endpoint. At a median follow-up of 35.3 months, there was no significant improvement in EFS with the addition of atezolizumab (HR 0.98, 95% CI = 0.71–1.36; *p* = 0.91). The addition of atezolizumab was also associated with increased toxicity, including higher rates of grade ≥3 TRAEs (22.7% versus 8.8%).

The negative results of ALBAN contrast with the positive findings from CREST and POTOMAC, demonstrating that the efficacy of checkpoint blockade in BCG-naïve NMIBC is potentially agent- and/or context-dependent [55]. Notably, in the CREST trial, EFS was significantly prolonged among patients with BCG-naïve CIS compared with those without (HR 0.53, 95% CI = 0.29–0.98); however, a similar EFS benefit in the CIS subgroup was not demonstrated in the POTOMAC (HR 1.01, 95% CI = 0.62–1.64) or ALBAN (HR 0.74, 95% CI = 0.43–1.28) trials [52,54,55]. Differences in treatment schedules and patient populations may partially explain discrepancies in these results. The duration of immune checkpoint inhibition exposure and BCG therapy varied. Patients received a longer duration of immune checkpoint inhibition in CREST relative to POTOMAC and ALBAN. Patients in ALBAN had just one year of BCG maintenance, in contrast to the two years given in CREST and POTOMAC. In addition, the ALBAN and POTOMAC trials enrolled a higher percentage of patients with CIS but a lower percentage of T1 and Ta disease compared to the CREST clinical trial. The definition of EFS in the ALBAN trial included low-grade NMIBC relapse, while low-grade NMIBC relapse was not included in the POTOMAC and CREST trials’ definitions of EFS. These key differences highlight the need for head-to-head trials to meaningfully compare the effectiveness of similar agents.

Following the success of KEYNOTE-057 in the setting of BCG-unresponsive NMIBC, KEYNOTE-676 (NCT03711032) is a phase III trial evaluating the efficacy of pembrolizumab combination with BCG versus BCG alone in high-risk BCG-naïve NMIBC (high-grade papillary Ta/T1 ± CIS). The primary endpoints are EFS and CR. The results have not yet been reported.

### 4.3. Frontline Systemic Immunotherapy Versus BCG

The phase IIb SunRISe-1 trial (NCT04640623) demonstrated encouraging activity of TAR-200 monotherapy, a novel intravesical gemcitabine delivery system, in the high-risk (CIS ± Ta/T1 papillary histology) BCG-unresponsive setting [54]. Building upon this success, the phase III SunRISe-3 trial (NCT05714202) is evaluating frontline use of systemic immunotherapy against standard-of-care intravesical BCG. In this open-label trial, 1135 patients with BCG-naïve high-risk (high-grade Ta, T1, and/or CIS) NMIBC are randomized 1:1:1 to receive TAR-200 alone, combination TAR-200 and cetrelimab, or BCG. The primary endpoint is event-free survival [60]. SunRISe-3 will help determine whether sustained-release intravesical gemcitabine, with or without immune checkpoint inhibition, offers a comparable or superior first-line option to BCG. This study remains active, with results pending.

## 5. Discussion

Despite the rapid expansion of immunotherapy options for NMIBC, intravesical BCG induction and maintenance remains the cornerstone of frontline management. Most novel agents and regimens investigated in phase II and III clinical trials have been positioned as salvage therapies for BCG-unresponsive disease or as adjuncts to BCG, and none have yet demonstrated superiority to intravesical BCG in the frontline setting. Nonetheless, approximately 30–50% of patients with high-risk NMIBC experience an incomplete response to BCG therapy [3], and ongoing global supply constraints further underscore the need for effective alternatives to BCG capable of achieving durable responses and bladder preservation.

A major barrier to defining the optimal sequencing and relative efficacy of these various immune-based therapeutic strategies is the absence of head-to-head clinical trials and the use of single-arm trials with variable designs. Cross-trial comparisons should be interpreted with caution due to differences in key trial characteristics, including patient population, eligibility criteria (i.e., CIS ± papillary versus papillary-only disease), sample size and statistical power, treatment administration and follow-up schedules, endpoint selection, and endpoint confirmation methods, among others. Even when these factors are aligned, residual heterogeneity limits the validity of cross-trial comparisons. Current FDA approvals for bladder-sparing immunotherapies in NMIBC are restricted to BCG-unresponsive disease with CIS ± papillary tumors. This underscores the importance of interpreting trial outcomes within their specific clinical and regulatory contexts.

With expanding treatment options that have distinct mechanisms of action and engage the innate-adaptive immune response continuum at varying points, it becomes increasingly difficult for urologists and oncologists to select therapies tailored to prior exposure or patterns of progression and recurrence. The long-term durability of these agents is unknown given the relatively short follow-up times for all these trials. Moreover, no randomized clinical trials have compared investigational treatments against upfront radical cystectomy, which is the current standard of care for BCG-unresponsive high-risk NMIBC. However, such a trial would be impractical to accrue patients. Without surgery, a true complete response (CR) visualized by cystoscopies, biopsies, urine cytology, and/or imaging may miss residual cancer, potentially overstating success. Although ethical considerations related to cystectomy morbidity and patient preferences may explain this gap, the lack of direct comparisons limits our ability to determine whether bladder-sparing therapies compromise long-term oncologic outcomes or delay necessary cystectomy until it becomes less effective or more morbid. Given that delayed cystectomy carries the potential risk of disease progression to unresectable or metastatic disease, careful patient selection for bladder-sparing approaches and close surveillance for treatment failure via repeated cystoscopies are essential for the early identification of non- or poor-responders who may require prompt transition to definitive surgical management.

Predictive biomarkers would enhance future investigations of immunotherapy in NMIBC. In the MIBC setting, increasing evidence suggests circulating tumor DNA (ctDNA) in the plasma after cystectomy may be a prognostic marker for disease recurrence and an indication for adjuvant immunotherapy [61,62]. If detectable, plasma ctDNA may have a role in NMIBC as well [63,64,65]. Given the likely lower burden of ctDNA in the plasma for localized disease, urinary tumor DNA (utDNA) may be a more effective method to evaluate minimal residual disease and to monitor risk of recurrence. For example, a secondary analysis of SWOG S1605 showed utDNA profiling at baseline and 3 months after atezolizumab could help identify patients with BCG-unresponsive NMIBC who were unlikely to benefit from atezolizumab [66]. Novel urinary biomarkers assessing protein expression, epigenetic patterns, and mRNA have shown potential utility in monitoring for high-grade disease recurrence. ADXBLADDER (Arquer Diagnostics Ltd., Sunderland, United Kingdom) uses ELISA to detect minichromosome maintenance protein 5 (MSM5), with higher MCM5 levels correlating with bladder cancer pathological staging [67]. Prospective studies report overall sensitivities of 45–73% and negative predictive values of 74–100% on initial diagnosis and surveillance superior to cytology [68]. Bladder EpiCheck (Nucleix, Rehovot, Israel) detects DNA methylation changes correlated with NMIBC progression, demonstrating sensitivity (62–90%) and negative predictive value (79–97%) superior to surveillance cytology [68,69]. Xpert Monitor BC^®^ (Cepheid, Sunnyvale, CA, USA) is an mRNA-based assay that has also shown high sensitivity (90.5%) and negative predictive value (95.3%) [70]. In patients who had undergone TURBT, there is evidence of an association between the elevation in blood levels of inflammatory markers, such as the platelet-to-lymphocyte ratio and systemic inflammation response index, and recurrence [71]. The incorporation of ctDNA, utDNA, inflammatory markers, and other potential liquid biopsy biomarkers into clinical trials evaluating therapy efficacy and recurrence of NMIBC warrants prospective validation. Hence, standardizing endpoints including standardized criteria for CR, investigating biomarker-driven stratification, and prioritizing comparative regimens will be crucial for translating the breadth of investigational therapies into consistent clinical algorithms.

As immune-based therapeutic strategies move earlier in the treatment paradigm, an additional challenge arises with regards to the delivery of these therapies and toxicity management. Combination treatments that utilize both intravesical and systemic immunotherapies could require combined skillsets of urologists and medical oncologists. Multidisciplinary care models integrating urology-led cystoscopic evaluation with oncology expertise in systemic therapy administration could address this but are not always practical. Moreover, the cost of these therapies should be considered and may not be widely available to most patients with NMIBC.

**Figure 1:**
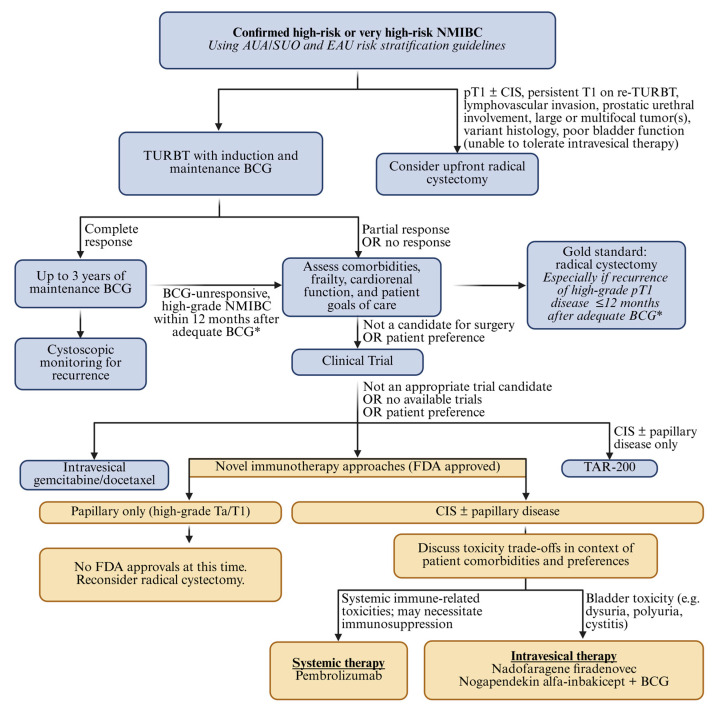
Simplified treatment algorithm for FDA-approved intravesical and systemic immunotherapy selection in NMIBC. * Per the most recent AUA/SUO guidelines [4], adequate BCG is defined as ≥5/6 induction doses plus either ≥2/3 maintenance doses or ≥2/6 doses of a second induction course. Figure created with Biorender.

Overall, recent advances in intravesical and systemic immunotherapy have expanded the therapeutic landscape for NMIBC and offer new opportunities for bladder preservation. The next challenge lies in transitioning from a proliferation of promising novel agents and regimens to evidence-based frameworks for clinical integration (Figure 1). Further investigation must address questions related to optimal sequencing relative to BCG, comparative efficacy between diverse treatment options, minimization of toxicities, and the safety of deferring cystectomy in favor of these various bladder sparing approaches. As these therapies continue to mature, coordinated multidisciplinary care and rigorous clinical evaluation will be essential to creating clinically meaningful and durable improvements in outcomes for patients with NMIBC.

## 6. Conclusions and Future Perspectives

Recent advances in intravesical and systemic immunotherapy have significantly expanded the treatment options for patients with NMIBC, both with and without CIS. While early studies have focused on patients with BCG-unresponsive disease who previously had limited bladder-sparing options, novel immunotherapies are being explored for frontline treatment. Expanded use of systemic immunotherapy, including subcutaneous therapies, innovative intravesical delivery systems, and novel immunomodulatory agents, have demonstrated encouraging durable response and reductions in recurrence rates, with several of these agents having received regulatory approval for clinical use. Together, these approaches not only broaden the bladder-sparing toolbox but also underscore the importance of mechanistically diverse immune activation in NMIBC management. As the field continues to evolve, defining optimal patient selection, sequencing therapies, assessing biomarkers, and integrating these agents into established care pathways will be essential. With ongoing clinical investigation and the development of new therapeutic modalities, the NMIBC treatment landscape is continually expanding with potential for additional personalized and effective strategies.

## Figures and Tables

**Table 1:** Included trials investigating the use of BCG for NMIBC.

Trial	Phase	Experimental Agent	Schedule	Total Enrollment	Population	Inclusion Criteria for Diagnosis	Median Follow-up	Notable Efficacy Outcomes	TRAEs ≥ G3 (%)
SWOG S1602 (NCT03091660) [20]	III	BCG (TICE vs. Tokyo-172)	6 weekly instillations ± 3-year maintenance ± intradermal priming	1000	BCG-naïve (high-grade Ta/T1/CIS)	Biopsy confirmed	NA	NA	NA
ANZUP 1301 (NCT02948543) [21]	III	BCG + intravesical mitomycin	6 weekly instillations + 10 monthly cycles; mitomycin administered weeks 3, 6, and 9, and months 3, 6, and 9	501	High-risk post-TURBT (Ta/pT1 ± CIS)	Biopsy confirmed	47 months	DFS at 24 months: HR 0.86 (95% CI = 0.64–1.14)	NA
BRIDGE (NCT05538663) [22]	III	Intravesical docetaxel/gemcitabine	6 weekly instillations of docetaxel/gemcitabine + monthly instillations for 2 years; control-arm BCG 6-week induction followed by maintenance for up to 3 years	870	BCG-naïve (high-grade Ta/T1/CIS)	Biopsy confirmed	NA	NA	NA

**Table 2:** Included trials investigating the intravesical administration of immunotherapy for NMIBC.

Trial	Phase	Experimental Agent	Schedule	Total Enrollment	Population	Inclusion Criteria for Diagnosis	Median Follow-up	Notable Efficacy Outcomes	TRAEs ≥ G3 (%)
ADVANCED-1/ADVANCED-2 (NCT05085990/NCT05951179) [23,24,25]	I/II	TARA-002	6 weekly instillations, followed by 3 weekly instillations every 3 months over an additional 15 months	131	BCG-naïve or BCG-unresponsive	Biopsy confirmed	NA	3-month CR: 38%	0
QUILT 3.032 (NCT03022825) [26,27]	II/III	Nogapendekin alfa-inbakicept + BCG	6 weekly instillations, followed by re-induction or maintenance up to 36 months	100	BCG-unresponsive (CIS ± Ta/T1)	Biopsy confirmed	NA (maximum >53 months)	CR: 71% (95% CI = 61.1–79.6%) Cystectomy-free rate at 36 months: 84%	3 (combined cohorts)
				80	BCG-unresponsive (high-grade Ta/T1)	Biopsy confirmed	NA	Median DFS: 25.3 months (95% CI 9.8–40.1 months) Cystectomy-free rate at 36 months: 82%	
NCT06829823 [28]	II	Nogapendekin alfa-inbakicept + BCG or gemcitabine	6 weekly instillations, followed by maintenance therapy up to 15 months	20	BCG-naïve (intermediate-risk Ta/T1)	Determined by investigator	NA	NA	NA
NCT02773849 [29]	III	Nadofaragene firadenovec	Instillations at months 3, 6, and 9	103	BCG-unresponsive (CIS ± Ta/T1)	Biopsy confirmed	19.7 months	3-month CR: 53.4% (95% CI = 43.3–63.3%) 12-month HGRFS: 24.3% (95% CI = 16.4–33.7%)	4 (combined cohorts)
				48	BCG-unresponsive (high-grade Ta/T1)	Biopsy confirmed	20.2 months	3-month HGRFS: 72.9% (95% CI = 58.2–84.7%) 12-month HGRFS: 43.8% (95% CI = 29.5–58.8%) median DOR: 12.35 months	
ABLE-22 (NCT06545955) [30]	II	Nadofaragene firadenovec ± gemcitabine/docetaxel or pembrolizumab	Instillations every 3 months up to 24 months	150	BCG-unresponsive (CIS ± high-grade Ta/T1)	Biopsy confirmed	NA	NA	NA
ABLE-32 (NCT06510374) [31]	III	Nadofaragene firadenovec	Instillations every 3 months up to 24 months	454	BCG-naïve (intermediate-risk)	Biopsy confirmed	NA	NA	NA
COMPARE-IT (NCT06929286) [32]	III	Nadofaragene firadenovec	Instillations every 3 months up to 12 months	125	BCG-unresponsive (high-grade)	Being treated for high-grade NMIBC with participating urologist	NA	NA	NA
BOND-002 (NCT02365818) [33]	II	Cretostimogene grenadenorepvec	6 weekly instillations, followed by maintenance therapy monthly up to 24 months	45	BCG-unresponsive (Ta/T1 or CIS ± Ta/T1)	Biopsy confirmed	NA	6-month CR: 47% (95% CI = 32–62%)	3% (dysuria), 1.5% (hypotension)
BOND-003 (NCT04452591) [34]	III	Cretostimogene grenadenorepvec + pembrolizumab	6 weekly instillations, followed by maintenance therapy up to week 97	190	BCG-unresponsive (Ta/T1 or CIS ± Ta/T1)	Biopsy confirmed	25.8 months	CR: 74.5% (95% CI = 65.4–82.4%)	0
CORE-001 (NCT04387461) [35]	II	Cretostimogene grenadenorepvec + pembrolizumab	6 weekly instillations, followed by maintenance therapy up to 24 months	35	BCG-unresponsive (CIS ± Ta/T1)	Biopsy confirmed	26.5 months	3-month CR: 83% (95% CI = 70.4–95.3%) 24-month CR: 51.4% (95% CI = 34.9–68.0%)	14.3
CORE-008 (NCT06567743) [36]	II	Cretostimogene grenadenorepvec	6 weekly instillations, followed by maintenance therapy up to 36 months	325	BCG-naïve or BCG-exposed (Ta/T1 or CIS ± Ta/T1)	Biopsy confirmed	NA	NA	NA
PIVOT-006 (NCT06111235) [37]	III	Cretostimogene grenadenorepvec	6 weekly instillations, followed by maintenance therapy up to 12 months	367	BCG-naïve (intermediate-risk)	Biopsy confirmed	NA	NA	NA
LEGEND (NCT04752722) [38]	I/II	Detalimogene voraplasmid	Instillations every 12 weeks for 4 cycles	21	BCG-unresponsive (CIS)	Biopsy confirmed	NA	3-month CR: 67% 6-month CR: 47%	0

**Table 3:** Included trials investigating the systemic administration of immunotherapy for NMIBC.

Trial	Phase	Experimental Agent	Schedule	Total Enrollment	Population	Inclusion Criteria for Diagnosis	Median Follow-up	Notable Efficacy Outcomes	TRAEs ≥ G3 (%)
KEYNOTE-057 (NCT02625961) [47,48]	II	Pembrolizumab	Every 3 weeks for up to 24 months	96	BCG-unresponsive (CIS ± papillary)	Biopsy confirmed	36.4 months	3-month CR: 41% (95% CI = 30.7–51.1%) 12-month CR: 19% Median CR: 16.2 months	13
				132	BCG-unresponsive (Ta/T1 papillary)	Biopsy confirmed	45.4 months	12-month DFS: 43.5% (95% CI = 34.9–51.9%) Median DFS: 7.7 months	15
SWOG S1605 (NCT02844816) [49]	II	Atezolizumab	Every 3 weeks for up to 12 months	129	BCG-unresponsive (high-grade Ta/T1 ± CIS)	Biopsy confirmed	41 months	27% CR for CIS group (did not meet prespecified efficacy threshold of 30% CR at 6 months in the CIS group) Median DOR: 17 months Durable response ≥12 months: 56% (95% CI = 34–77%)	16
ADAPT-BLADDER (NCT03317158) [50]	I	Durvalumab ± BCG or EBRT	Every 3 weeks for up to 8 cycles	28	BCG-unresponsive (high-grade Ta/T1 ± CIS)	Biopsy confirmed	20.6 months	Durvalumab monotherapy: 3-month CR: 33% 12-month CR: 0% Durvalumab + BCG: 3-month CR: 85% 12-month CR: 73% Durvalumab + EBRT 3-month CR: 50% 12-month CR: 33%	11
DURANCE (NCT04106115) [51]	Ib/II	Durvalumab + S-488210/S-488211	Durvalumab every 4 weeks for up to 7 cycles; vaccine weekly for 6 weeks, then every other week for 9 weeks	14	BCG-unresponsive or BCG-intolerant (Ta/T1/CIS)	Biopsy confirmed	NA	3-month DFS: 71% 6-month DFS: 57%	0
POTOMAC (NCT03528694) [52]	III	Durvalumab + BCG	Durvalumab every 4 weeks for 13 cycles; 6 weeks of BCG induction + maintenance at 3, 6, 12, 18, and 24 months	1018	BCG-naïve (high-grade Ta/T1 ± CIS)	Biopsy confirmed	60.7 months	24-month DFS: 86.5% (95% CI = 82.2–89.8%)	21
PATAPSCO (NCT05943106) [53]	IIIb	Durvalumab + BCG	Durvalumab every 4 weeks for 14 cycles; BCG induction + maintenance up to 24 months	100	BCG-naïve (Ta/T1 ± CIS)	Biopsy confirmed	NA	NA	NA
CREST (NCT04165317) [54]	III	Sasanlimab + BCG	Sasanlimab every 4 weeks for up to 25 cycles; BCG induction ± second induction, maintenance up to 3 years	1055	BCG-naïve (high-grade Ta/T1 ± CIS)	Biopsy confirmed	36.3 months	Addition of sasanlimab reduced the risk of events by 32% relative to BCG alone (HR 0.68, 95% CI = 0.49–0.94)	29.1
ALBAN (NCT03799835) [55]	III	Atezolizumab + BCG	Atezolizumab every 3 weeks for 1 year; BCG induction (6 weeks) + maintenance at 3, 6, and 12 months	517	BCG-naïve (Ta/T1 ± CIS)	Biopsy confirmed	35.3 months	No EFS benefit vs BCG alone (HR 0.98, 95% CI = 0.71–1.36)	22.7
KEYNOTE-676 (NCT03711032) [56]	III	Pembrolizumab + BCG	Pembrolizumab every 3 weeks for up to 35 cycles; BCG induction + maintenance through 36 months	1405	BCG-naïve (high-grade Ta/T1 ± CIS)	Biopsy confirmed	NA	NA	NA
SunRISe-1 (NCT04640623) [57]	IIb	TAR-200 ± cetrelimab; cetrelimab alone	TAR-200 every 3 weeks for 24 weeks, then every 12 weeks through week 99; cetrelimab every 3 weeks through week 78	218	BCG-unresponsive (CIS ± Ta/T1); separate HG papillary-only cohort	Biopsy confirmed	TAR-200 (CIS ± Ta/T1): 20.2 months TAR-200 (papillary only): 12.8 months TAR-200 + cetrelimab (CIS ± Ta/T1): 33.4 months Cetrelimab (CIS ± Ta/T1): 29.2 months	TAR-200 CR: 82.4% (95% CI = 72.6–89.8%) TAR-200 median DOR: 25.8 months Combination CR: 67.9% (95% CI = 53.7–80.1%) Cetrelimab CR: 46.4% (95% CI, 27.5–66.1%)	12.9 (TAR-200)
SunRISe-3 (NCT05714202) [58]	III	TAR-200 ± cetrelimab or BCG	TAR-200 every 3 weeks; BCG induction (6 weeks) + maintenance at weeks 12, 24, 48, 72, and 96; cetrelimab ever 3 weeks through week 51	1135	BCG-naïve (high-grade Ta/T1 ± CIS)	Biopsy confirmed	NA	NA	NA

## Data Availability

No new data were created for this work.

## Abbreviations

The following abbreviations are used in this manuscript:

AUA	American Urological Association
BCG	Bacillus Calmette-Guérin
CG0070	Cretostimogene grenadenorepvec
CI	Confidence interval
CR	Complete response
CIS	Carcinoma in situ
DFS	Disease-free survival
EAU	European Association of Urology
EBRT	External beam radiation therapy
FDA	Food and Drug Administration
GM-CSF	Granulocyte macrophage colony-stimulating factor
HR	Hazard ratio
INFα2b	Human interferon alpha-2b
MIBC	Muscle-invasive bladder cancer
N-803	Nogapendekin alfa inbakicept
NMIBC	Non-muscle-invasive bladder cancer
RCT	Randomized controlled trial
RIG-I	Retinoic acid-inducible gene I
SUO	Society of Urologic Oncology
TNF	Tumor necrosis factor
TRAIL	Tumor necrosis factor-related apoptosis-inducing ligand
TRAEs	Treatment-related adverse effects
TURBT	Transurethral resections of bladder tumors
